# Supplementary material for: Rail-dbGaP: analyzing dbGaP-protected data in the cloud with Amazon Elastic MapReduce
Source: Bioinformatics. 2016 Apr 21;32(16):2551–3. doi: 10.1093/bioinformatics/btw177 (PMC4978928; doi:10.1093/bioinformatics/btw177)
Supplement: Supplementary Data [file supp_32_16_2551__index.html]

Rail-dbGaP: analyzing dbGaP-protected data in the cloud with Amazon Elastic MapReduce — Rail-dbGaP: analyzing dbGaP-protected data in the cloud with Amazon Elastic MapReduce — Rail-dbGaP: analyzing dbGaP-protected data in the cloud with Amazon Elastic MapReduce — Supplementary Data 

# Rail-dbGaP: analyzing dbGaP-protected data in the cloud with Amazon Elastic MapReduce

## Supplementary Data

files

- Supplementary Data - pdf file
- Supplementary Data - pdf file
