# Supplementary material for: Rail-dbGaP: analyzing dbGaP-protected data in the cloud with Amazon Elastic MapReduce
Source: Bioinformatics. 2016 Apr 21;32(16):2551–3. doi: 10.1093/bioinformatics/btw177 (PMC4978928; doi:10.1093/bioinformatics/btw177)
Supplement: Supplementary Data [file supp_btw177_rail-dbgap_manual.pdf]

The Rail-dbGaP protocol

Analyzing dbGaP-protected data from the Sequence Read Archive using Amazon Elastic MapReduce

v0.1.0

MIT-licensed; see LICENSE.

Table of Contents

1. MapReduce and Elastic MapReduce
2. Rail-dbGaP protocol specification
3. Setting up Amazon Web Services
4. Studying k-mers in dbGaP-protected samples with EMR
5. A note on TCGA data and other data sources
6. Helpful notes for administrators

The National Institutes of Health (NIH) maintains security requirements and recommmendations for analyzing controlled-access genomic data, including dbGaP-protected data. Rail-dbGaP is a protocol described in this preprint for securely analyzing dbGaP-protected genomic data from the Sequence Read Archive (SRA) in the cloud with Amazon Elastic MapReduce (EMR) in a manner compliant with NIH guidelines. The protocol is implemented in Rail-RNA, software for scalable analysis of many hundreds of RNA sequencing (RNA-seq) samples. A step-by-step guide for setting up Rail-RNA to analyze dbGaP-protected RNA-seq data is provided in the Rail documentation here; the present document contains a technical specification of the Rail-dbGaP protocol and walks the user through an example implementation that counts the number of input dbGaP samples on SRA (i.e., SRA run accession numbers) in which each k-mer present in at least one read from among the samples appears. A preprint describing the Rail-dbGaP protocol is available here.

MapReduce and Elastic MapReduce

The MapReduce programming model divides a problem into a sequence of alternating computation and aggregation steps. Each step is performed by distributing independent tasks across workers in a cluster of computers. Elastic MapReduce (EMR) is a Hadoop-based implementation of MapReduce especially for a cluster of Elastic Compute Cloud (EC2) instances, or virtualized computers, on Amazon Web Services, a commercial cloud provider. EMR reads input from the web and/or Simple Storage Service (S3), Amazon's cloud storage service, and writes its output back to S3.

Rail-dbGaP protocol specification

Rail-dbGaP secures an EMR cluster so it is compliant with NIH guidelines as follows. (See Figure 1 of the preprint for an illustration. The sections Setting up Amazon Web Services and Studying k-mers in dbGaP-protected samples with EMR put together protocol elements to show their implementation explicitly.)

1. **The cluster is created within a subnet of a Virtual Private Cloud (VPC)**, a logically isolated unit of the cloud providing a private network and firewall. The connection with S3 is via a VPC endpoint, which ensures that data transferred never leaves the data center. Each instance has a public IP. The VPC is set up by creating a stack with the CloudFormation template `cloudformation/dbgap.template` .

2. **Inbound traffic to the cluster is restricted via security groups.** A security group is essentially a stateful firewall. A master security group for the master instance and a worker security group for worker instances prevent initiation of any connection to the cluster except by essential web services. These web services correspond to particular IPs and ports,

and the most restrictive sets for master and worker instances are configured automatically. SSH access to the cluster is also restricted: the only interaction between user and cluster is via the EMR interface, which presents progress information through the essential web services. Security groups are also set up by creating a stack with the CloudFormation template `cloudformation/dbgap.template` . Master and worker instances must be associated with security groups when creating the EMR cluster.

3. **Data are encrypted at rest.** During cluster setup, before any sensitive data has reached the cluster, each instance runs the preliminary script (i.e., bootstrap action) `bootstraps/encrypt_local_storage.sh` that uses Linux Unified Key Setup (LUKS) to create an encrypted partition with a keyfile. The key is randomly generated on each instance and never exposed to the user. Temporary files, the Hadoop distributed file system, and buffered output to the cloud storage service are all configured to reside on the encrypted partition via symbolic links. (See line 128 of `bootstraps/encrypt_local_storage.sh` .) Files written to cloud storage are also encrypted; Amazon S3 uses AES256. This is enforced by the creation of a bucket with a policy (i.e., rules governing user access to the bucket) barring uploads that do not turn on server-side encryption in the dbGaP template `cloudformation/dbgap.template` and by setting the EMRFS configuration parameter `fs.s3.enableServerSideEncryption=true` .
4. **Data are encrypted in transit.** Worker instances download dbGaP data using SRA Tools, ensuring encryption of data transferred from dbGaP to the cluster. AWS enables Secure Sockets Layer (SSL) by default for transfers between cloud storage and the cluster as well as between cloud storage service and compliant local storage to which an investigator saves results.
5. **Identities are managed to enforce the principle of least privilege.** The principle of least privilege prescribes users have only the privileges required to perform necessary tasks. In the Rail-dbGaP protocol, an administrator grants the user only those privileges required to run Hadoop programs on EMR clusters. The administrator uses multi-factor authentication and constrains the user to set up a password satisfying NIH's requirements listed among security best practices (minimum of 12 characters, no complete dictionary words, etc.) On AWS, an account administrator configures an Identity and Access Management (IAM) user expressly for running Hadoop jobs and retrieving results from S3, and the password rules described above are enforced. This is achieved as described below in Setting up Amazon Web Services.
6. **Audit logs are enabled.** These record logins and actions taken by the user and on the user's behalf, including API calls made by processes running on the cluster. On AWS, audit logs take the form of CloudTrail logs stored in encrypted S3 buckets. They are enabled when a stack is created with the dbGaP template `cloudformation/dbgap.template` .

Setting up Amazon Web Services

The steps below create a new AWS IAM account especially for analyzing dbGaP-protected data. To perform these steps, both user and AWS site administrator should be available. (For many investigators, user and administrator will be the same person.) It is recommended that they are physically together to minimize passing of credentials. Before continuing, the user should install the AWS Command Line Interface (CLI). Optionally, the user may also have requested access to some dbGaP-protected sample on the Sequence Read Archive (SRA) and received a key file with an `ngc` extension.

Set up an administrator account (administrator)

These steps should be performed if the site administrator is new to AWS.

1. Navigate to http://aws.amazon.com/free in your web browser.
2. Click **Create a free account**.
3. Check the **I am a new user** box and and continue to follow the instructions to create your new account. You'll enter, for example, contact and payment information. Note that the **Basic** level of service is sufficient for our purposes.
4. Make a note of your account number.
   i. Log into the AWS console using the new account's email address and password.
   ii. Click on the arrow next to your user name in the gray banner at the top of the page.
   iii. Select **My Account**, and the **Account Id** will be displayed at the top of the page.

5. Secure the account
   i. Log into the AWS console using the new account's email address and password.
   ii. Open the **Identity and Access Management** page.

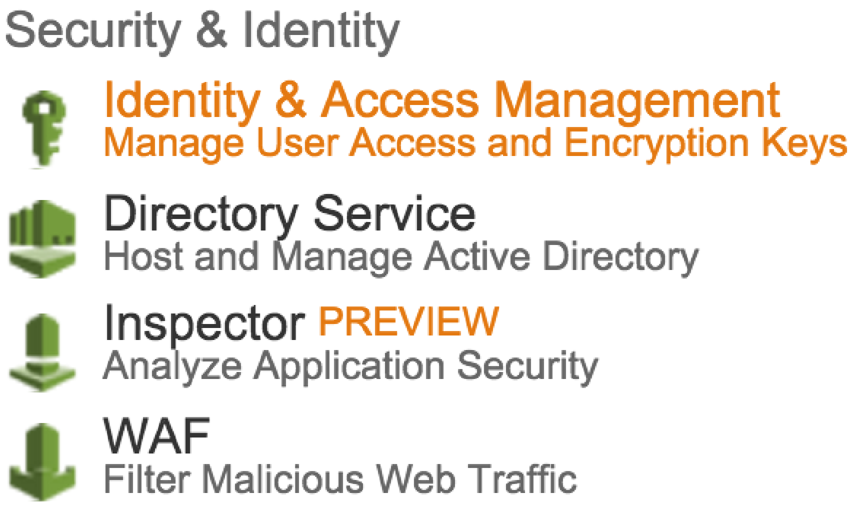

   iii. Under **Security Status**, click **Activate MFA on your root account**, then click **Manage MFA**, and follow the instructions to enable multi-factor authentication. We use a virtual MFA device (smartphone) with Google Authenticator.

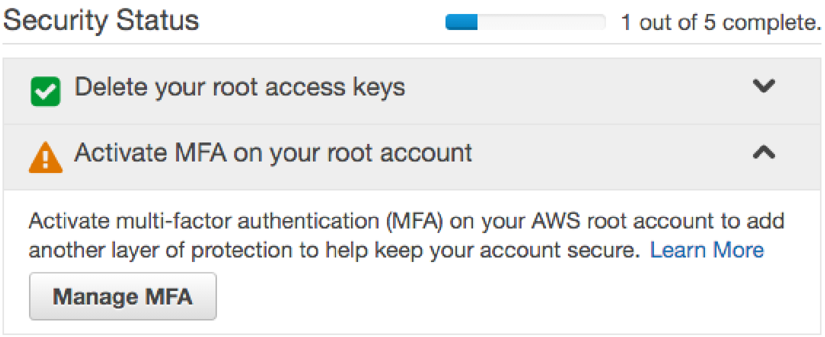

   iv. Under **Apply an IAM password policy**, click **Manage Password Policy**.

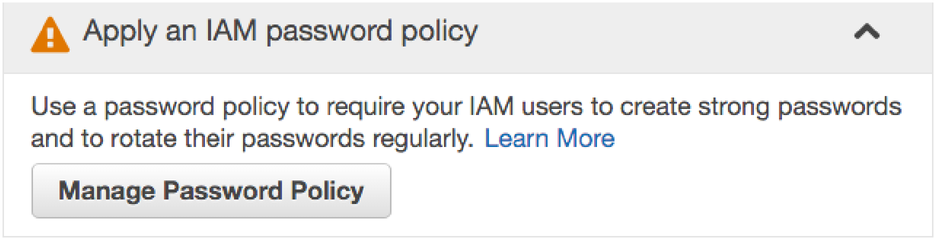

   Configure the password policy according to the requirements mentioned in the NIH Security Best Practices for Controlled-Access Data Subject to the NIH Genomic Data Sharing (GDS) Policy. This usually entails the following, but please note that your institution may impose more stringent requirements:

   - Requiring a minimum password length of 12
   - Requiring at least one uppercase letter
   - Requiring at least one lowercase letter
   - Requiring at least one number
   - Requiring at least one non-alphanumeric character
   - Enable password expiration after 120 days

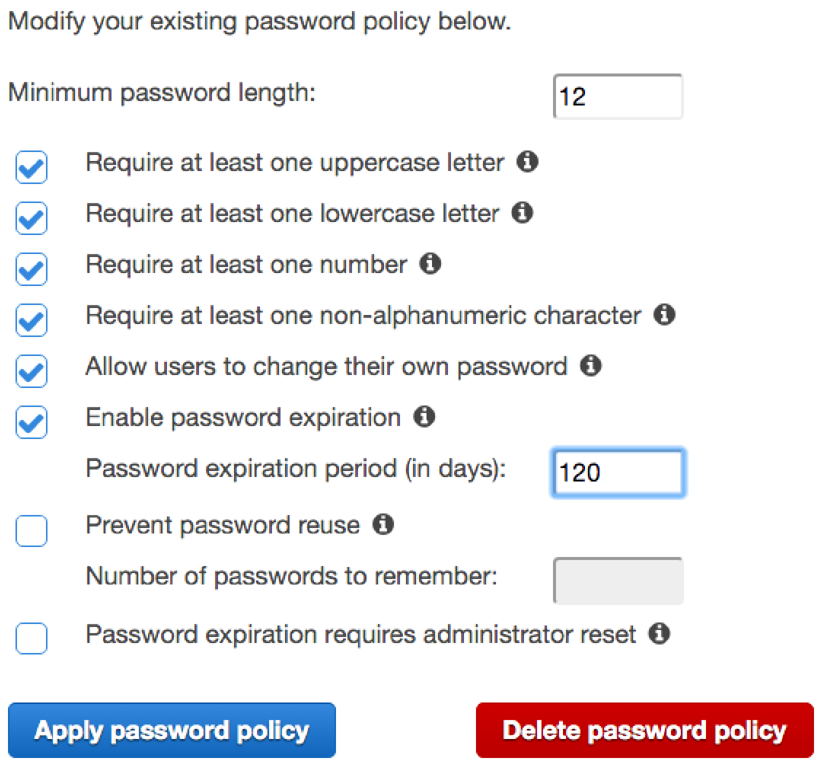

v. Click **Apply password policy**.

Set up a new IAM user (administrator & user)

During this process, it is best for the account administrator to sit with the user to minimize passing credentials.

1. *Administrator:* create a new IAM user.

   i. From the new user's computer, log into the AWS Console and select **Identity and Access Management**.
   ii. Click **Users** on the left pane, then **Create New Users** on the right pane.

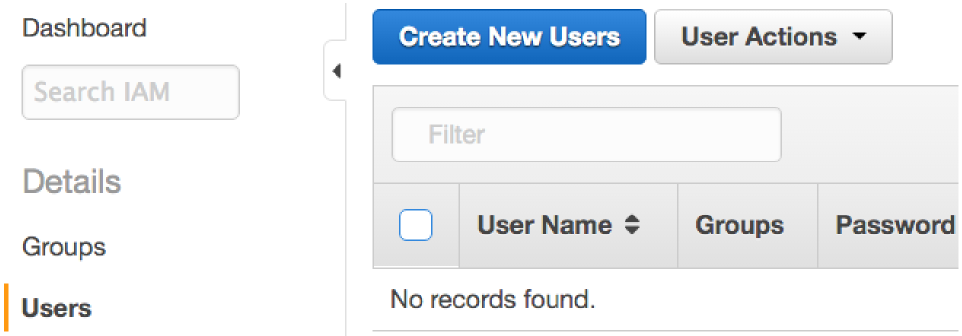

   iii. Enter the new user's username. We call the new user **dbgapuser** in the screenshot. Check the **Generate an access key for each user** checkbox, and click **Create**.

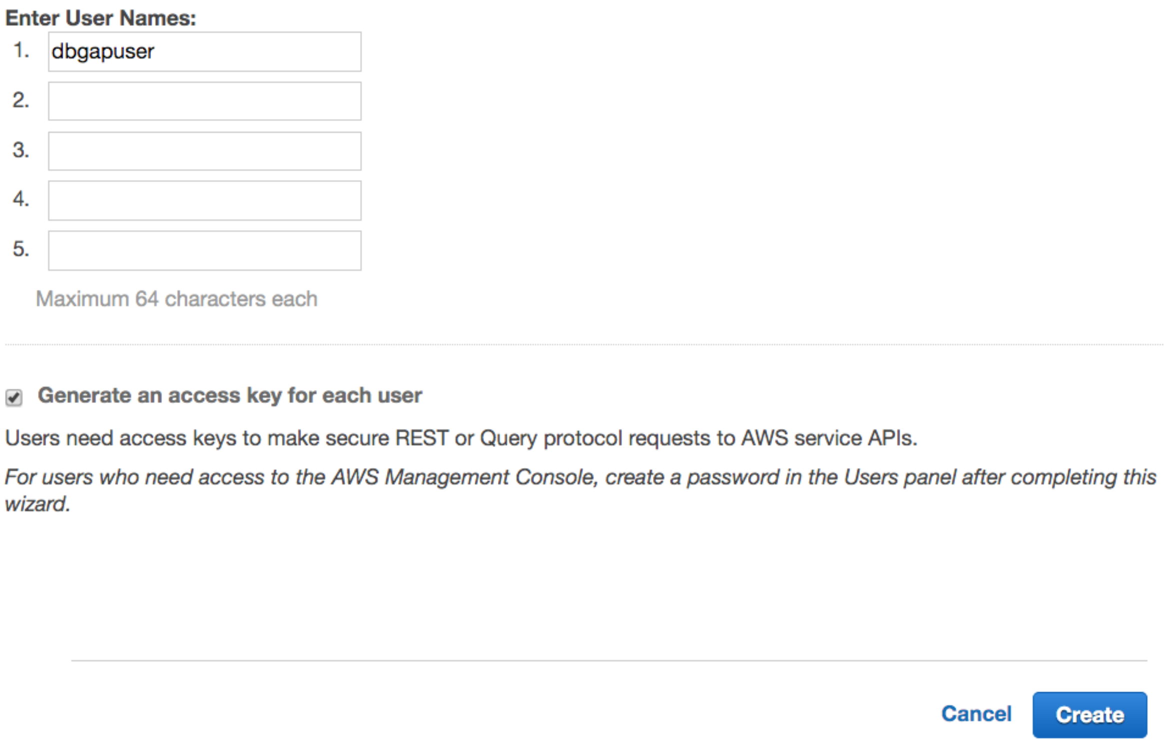

iv. Click **Download Credentials**. These credentials (*credentials.csv*) include the AWS Access Key ID and AWS Secret Access Key. It is recommended that the file containing the credentials be made readable only by the user immediately. The credentials should never be shared, intentionally or inadvertently, with anyone else.

2. *User:* register credentials with the AWS CLI by entering

```
aws configure --profile dbgap
```

at a terminal prompt on the user's computer. Enter the AWS Access Key ID, AWS Secret Access Key, and a default region as prompted. We recommend using `us-east-1` because its connection to dbGaP-protected data on SRA appears to be fastest. A default output format need not be specified. Now the new user can issue AWS API calls via the AWS CLI. *It is recommended that credentials file that was just downloaded is now deleted.*

3. *Administrator:* Set user's password.

i. Return to the AWS Console, again click **Identity and Access Management**, again click **Users** on the left sidebar, and select the new user. Under **User Actions**, click **Manage Password**.

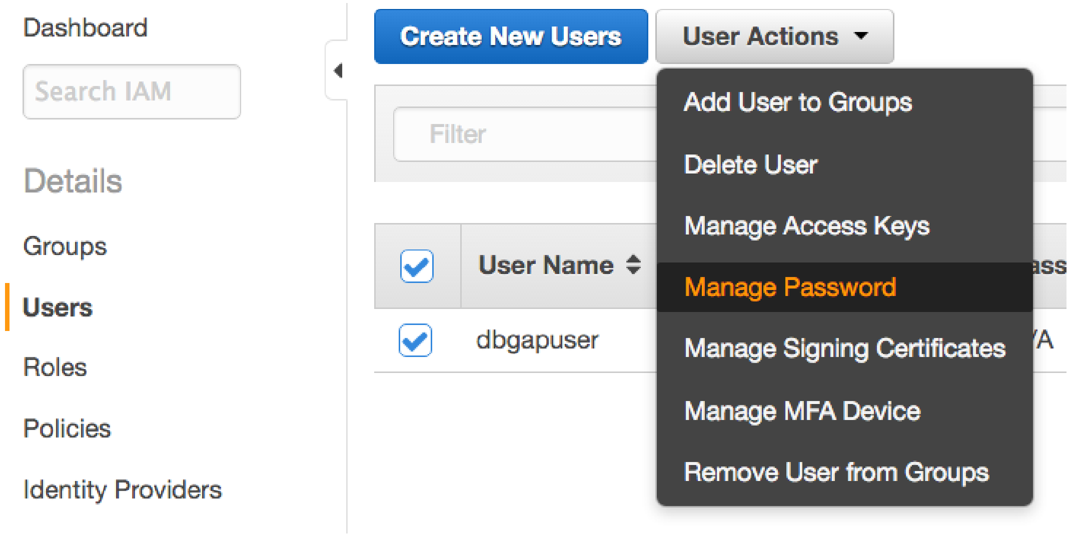

ii. Select **Assign an auto-generated password**, check the **Require user to create a new password at next sign-in** box, and click **Apply**.

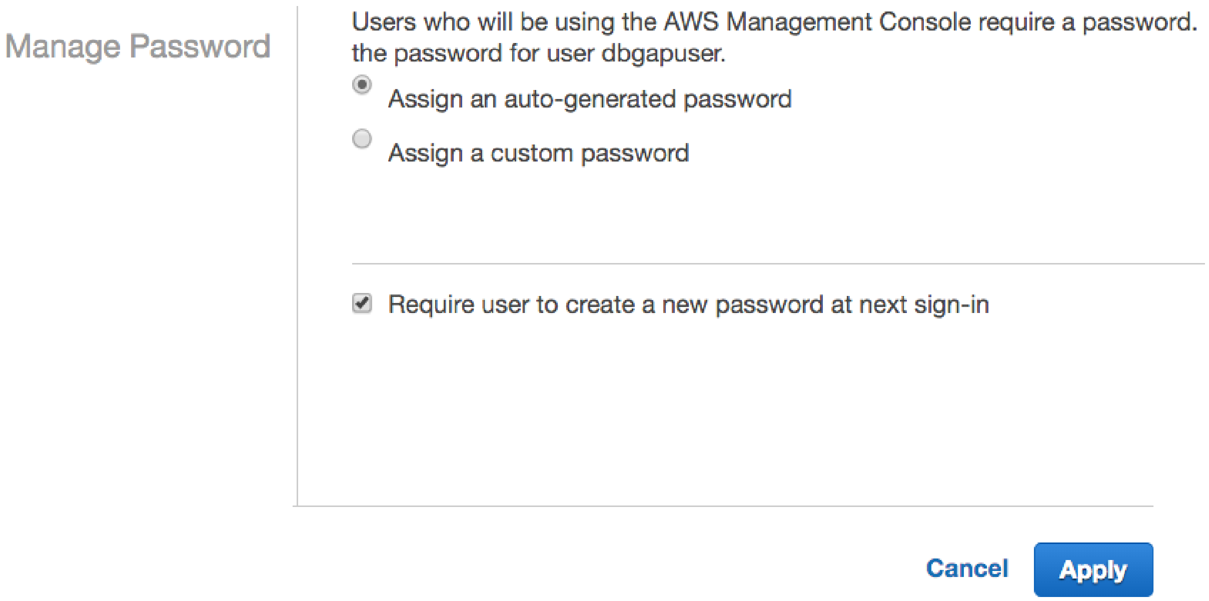

iii. Click **Download Credentials**. The new credentials file *credentials (2).csv* contains the username, the auto-generated password, and the URL for the account-specific login page.

4. *User:* navigate to the login page URL from *credentials (2).csv*, log in, and change the password as prompted.

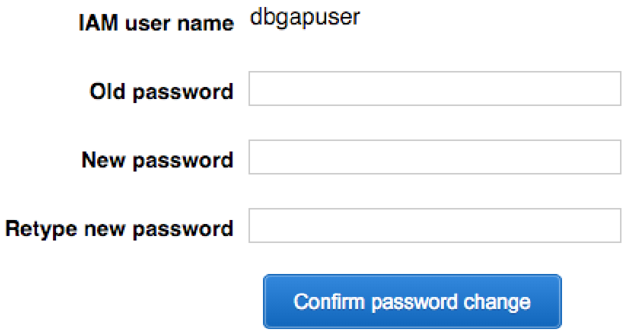

Create a secure CloudFormation stack (administrator)

CloudFormation facilitates creation and management of a group of related AWS resources. Rail-dbGaP provides a CloudFormation template for creating a Virtual Private Cloud (VPC) with a single public subnet. An EMR job flow that analyzes dbGaP data should be launched into this subnet. The VPC is supplemented by several security features, including

- a VPC endpoint for S3, which ensures that the connection between the Elastic MapReduce cluster and S3 is private.
- security groups that block all inbound traffic to the cluster from the internet except from the Elastic MapReduce webservice.
- the creation of a secure bucket on S3 into which all output should be written when operating on dbGaP-protected data. The bucket has an attached policy barring uploads that do not have server-side encryption (AES256) turned on.
- CloudTrail logs recording AWS API calls. These are written to the secure bucket.

The administrator should grab the latest version of the template here. Implement it by following these steps. (If the administrator already has CloudTrail turned on, they may not work, causing a rollback. An administrator satisfied with their CloudTrail configuration may instead want to use this alternative CloudFormation template, which creates the VPC but does not attempt to set up CloudTrail.)

1. Click **CloudFormation** in the AWS console, making sure the region in the upper-right corner of the screen is the same

as the user's default region (typically `us-east-1`, i.e., N. Virginia).

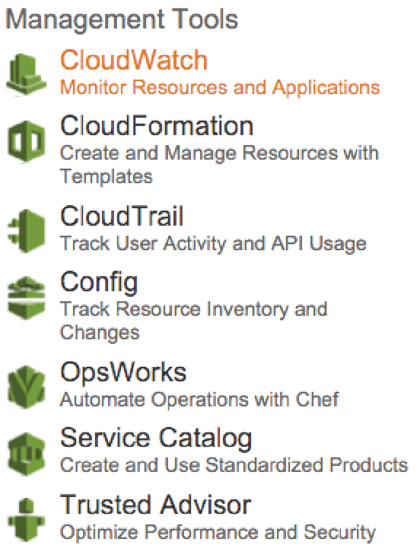

2. Click **Create Stack**.

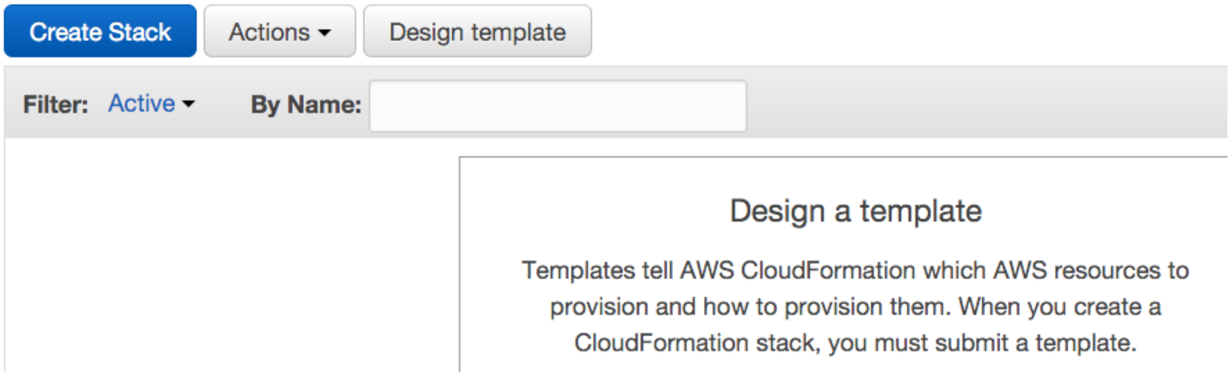

3. Under **Choose a template**, opt to upload `dbgap.template` to Amazon S3, and click **Next**.

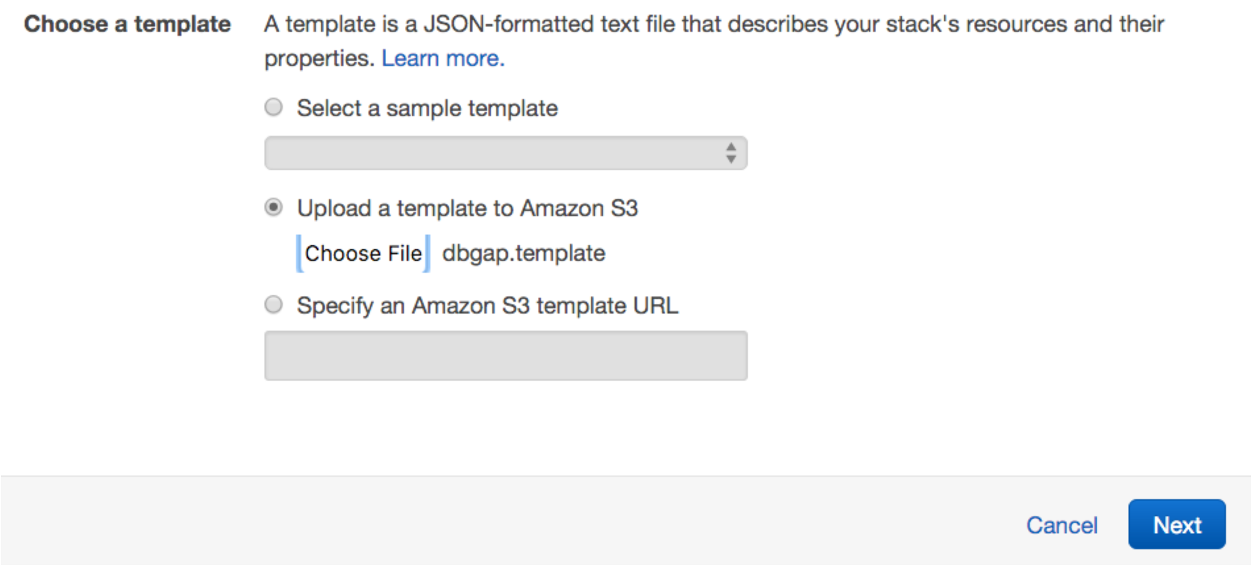

4. On the next screen:
   - Next to **Stack name**, write "dbgap".
   - Next to **Parameters**, let the user type the name of a secure bucket into which they will write all output. The bucket name should not have been taken by any other S3 user.

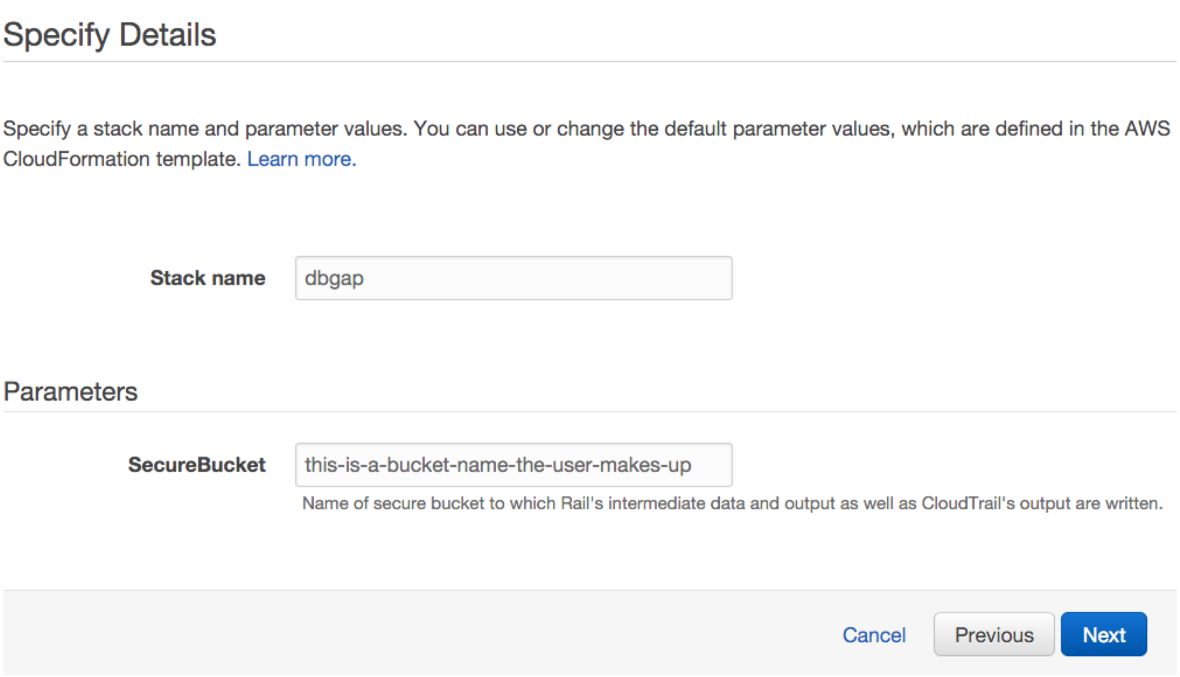

5. Click **Next** and **Next** again, then click **Create** and wait for the stack creation to complete. The status message "CREATE_COMPLETE" will soon appear next to "dbgap" on the list of stacks.

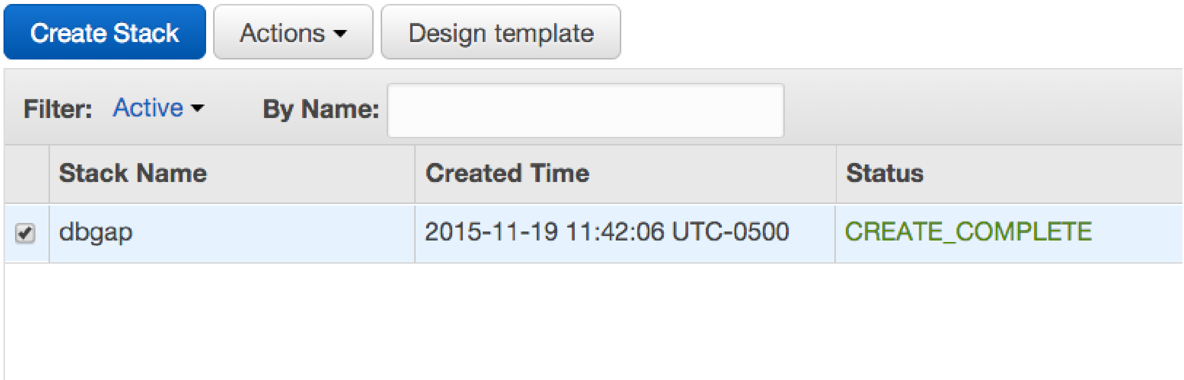

The best defense is a good offense, and you are encouraged to monitor traffic to clusters launched by the user. You may want to explore turning on VPC flow logs and CloudWatch alarms for suspicious activity.

Delegate Elastic MapReduce and CloudFormation authorites to the new IAM user (administrator)

The new IAM user still needs sufficient privileges to use Elastic MapReduce.

1. Return to the AWS Console, again click **Identity and Access Management**, but now click **Policies** on the left sidebar.

2. Click **Create Policy**, then select **Create Your Own Policy**. (You may need to click **Get Started** first.)

    i. Under **Policy Name**, enter "UseExistingEMRRoles".

    ii. Under **Policy Document**, paste the following.

```
{
    "Version": "2012-10-17",
    "Statement": [
        {
            "Effect": "Allow",
            "Action": [
                "iam:GetInstanceProfile",
                "iam:GetRole",
```

```
                "iam:PassRole"
            ],
            "Resource": "*"
        }
    ]
}
```

iii. Click **Create Policy**.

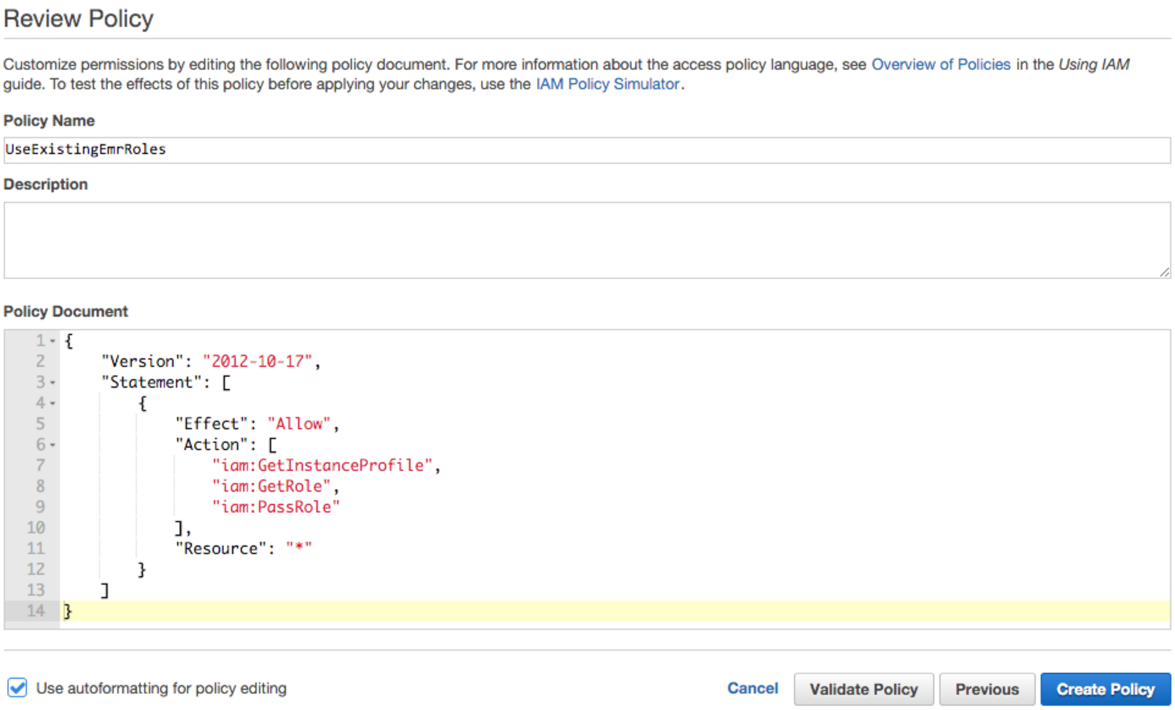

3. Now click **Users** in the left pane, select the new IAM user, and click the **Permissions** tab.

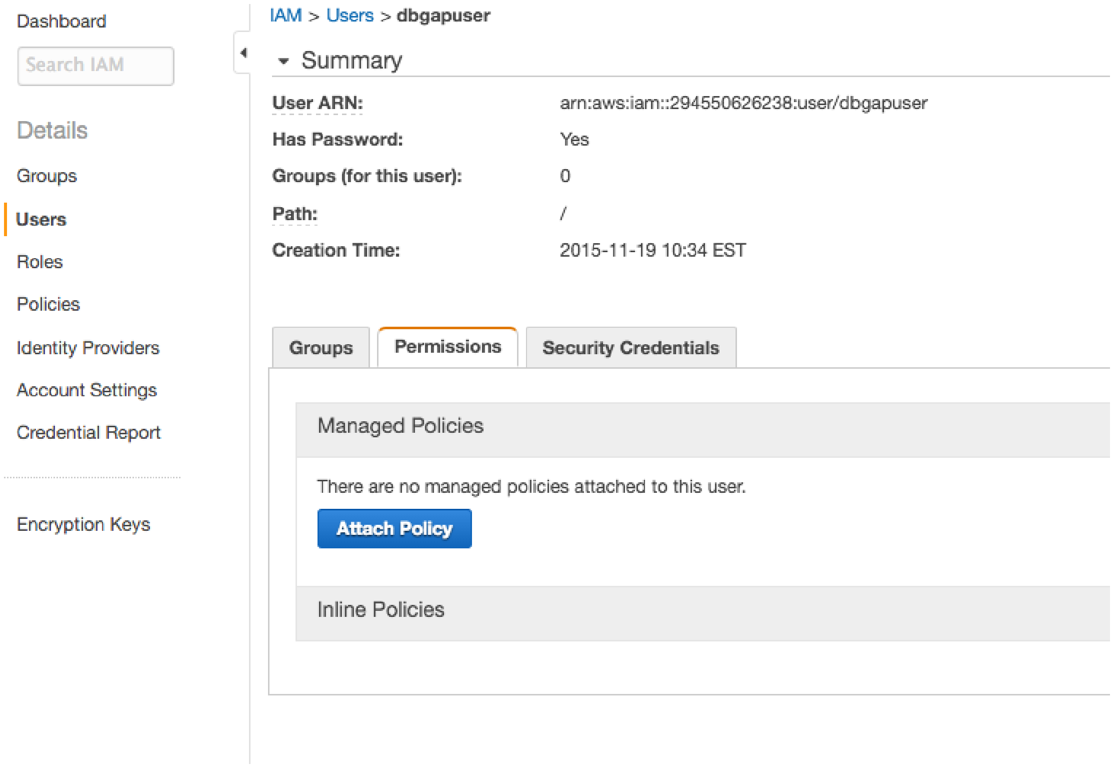

4. Click **Attach Policy**, and select the `AWSCloudFormationReadOnlyAccess` , `AmazonElasticMapReduceFullAccess` , and `UseExistingEMRRoles` policies. Then click Attach Policy.

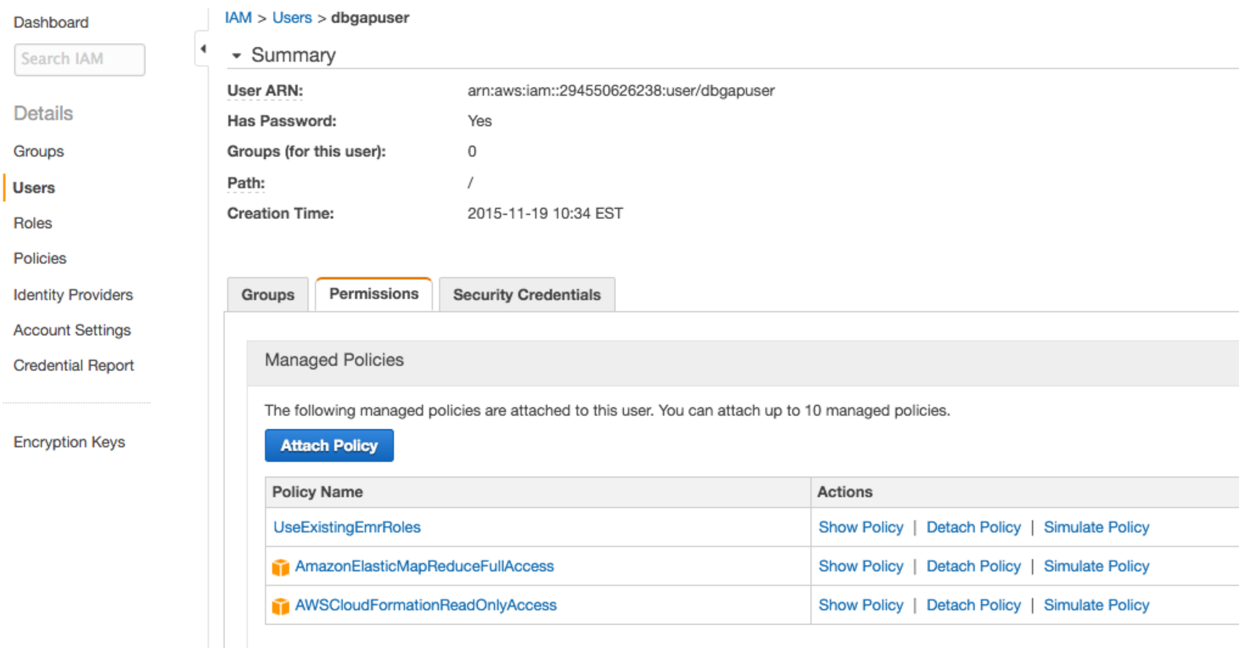

Different policies including only some of the permissions from these may be included, but note that the user must be able to: (1) launch Elastic MapReduce clusters into the VPC from the secure dbGaP CloudFormation stack created by the administrator above, and (2) read and write to the secure S3 bucket created by the administrator on behalf of the user.

Set up default EMR roles (administrator & user)

1. *Administrator:* follow these instructions to create default roles for Elastic MapReduce.
2. *User:* run

   ```
   aws emr create-default-roles --profile dbgap
   ```

   to retrieve the default Elastic MapReduce roles created by the administrator.

Studying k-mers in dbGaP-protected samples with EMR

This section reviews the implementation of an EMR pipeline that ingests dbGaP-protected data and counts the number of samples in which each *k*-mer found in least one read across samples appears. dbGaP support has kindly provided a dataset composed of public RNA-seq samples from 1000 Genomes exclusively for testing secure cloud-based pipelines. Its project accession number on SRA is SRP041052, and the steps below use three samples from it.

Assume the secure bucket created during AWS setup is at `s3://rail-dbgap-secure` . The following is performed on the user's computer, where the AWS CLI was installed.

1. Download the dbGaP repository key for the test data at `ftp://ftp.ncbi.nlm.nih.gov/sra/examples/decrypt_examples/prj_phs710EA_test.ngc` . Upload the key to S3 securely with the AWS CLI by entering

   ```
   aws s3 cp /path/to/prj_phs710EA_test.ngc s3://rail-dbgap-secure/test/prj_phs710EA_test.ngc --profile dbgap --sse
   ```

   It is recommended that you delete the key from your computer with

   ```
   rm /path/to/prj_phs710EA_test.ngc
   ```

2. Using a text editor, create a script `copy_files_to_node.sh` with the following contents.

```
#!/usr/bin/env bash
set -ex
aws s3 cp s3://rail-dbgap-secure/test/prj_phs710EA_test.ngc /mnt/space/DBGAP.ngc
aws s3 cp s3://rail-emr/step.sh /mnt/space/step.sh
```

   Copy the script to S3 as follows.

```
aws s3 cp /path/to/copy_files_to_node.sh s3://rail-dbgap-secure/test/ --profile dbgap --sse
```

   This script will be a bootstrap action that (1) securely transfers the key to each EC2 instance and (2) copies the mapper script to each instance. The mapper script is discussed below.

3. Download this list of three SRA run accession numbers from test dbGaP project SRP041052. Copy it to S3 as follows

```
aws s3 cp /path/to/manifest.txt s3://rail-dbgap-secure/test/ --profile dbgap --sse
```

4. Navigate to the login page URL from *credentials (2).csv*. The AWS console should appear immediately or after you log in with the credentials from this CSV.
5. Click **EMR**, then **Create cluster**, and finally **Go to advanced options**.

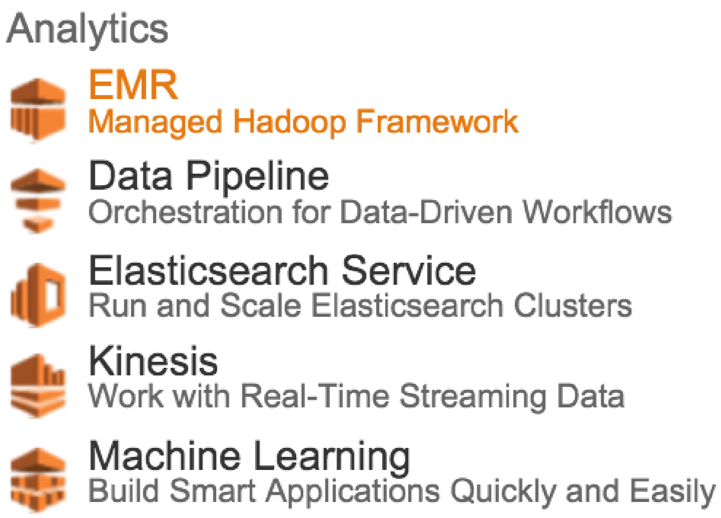

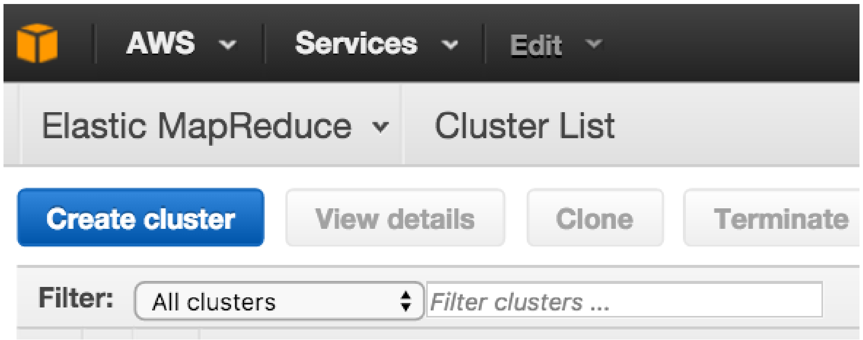

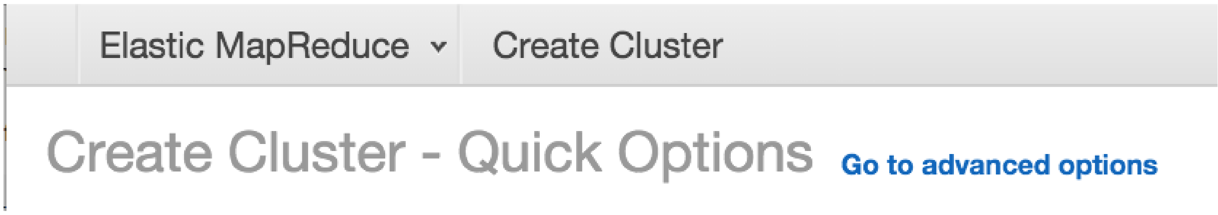

6. Under **Software Configuration**, ensure the Vendor Amazon is selected, and select 3.11.0 under Release. Deselect all

software except Hadoop 2.4.0.

7. Under **Add steps (optional)**, select **Streaming program** next to **Step type**, and click **Configure**.

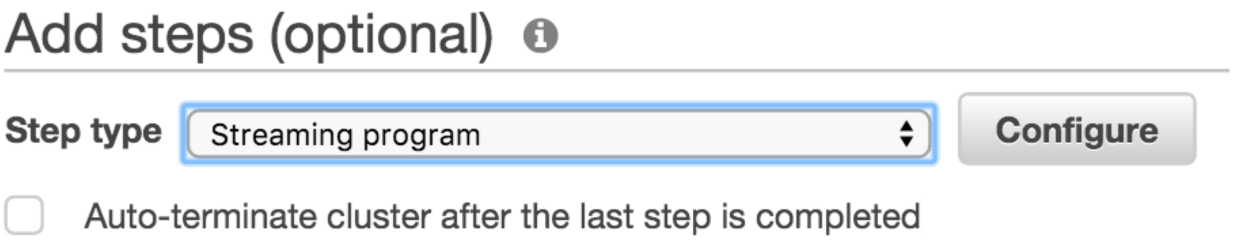

8. Next to **Name**, type "Count number of samples in which each k-mer appears".

9. Next to **Mapper**, enter

```
bash /mnt/space/step.sh
```

This is the script here, and it is reproduced below.

```
#!/usr/bin/env bash
set -ex;
cut -f2 | { IFS= read -r SRR;
KMERSIZE=21;
cd /mnt/space/sra_workspace/secure;
fastq-dump ${SRR} --stdout -X 10000 \
  | bioawk -v kmersize=${KMERSIZE} -v srr=${SRR} -c fastx \
    '{
        for (i=1; i<=length($seq)-kmersize; i++) {
            revcompsubseq = substr($seq, i, kmersize);
            subseq = revcompsubseq;
            revcomp(revcompsubseq);
            if (revcompsubseq < subseq) {
                print "UniqValueCount:" revcompsubseq "\t" srr;
            } else {
                print "UniqValueCount:" subseq "\t" srr;
            }
        }
    }'
}
```

It describes a mapper that uses SRA Tools `fastq-dump` to grab an input sample from SRA and Heng Li's bioawk to extract k-mers (for k=21) from its read sequences to print either the k-mer or its reverse complement, whichever is first in lexicographic order. `UniqValueCount` refers to how aggregation should be performed by Hadoop Streaming: the reducer described in the next step will count the number of unique run accession numbers associated with a given k-mer. The command-line parameter `-X 10000` of `fastq-dump` grabs only the first 10,000 reads of each sample for the purpose of demonstration only. Both SRA Tools and bioawk will have to be installed by bootstrap scripts, which are configured in later steps.

10. Next to **Reducer**, enter

```
aggregate
```

This allows the `UniqValueCount` s output by the mappers to be interpreted properly. See this page for more information on the Hadoop Aggregate package.

11. Next to **Input S3 location**, enter

```
s3://rail-dbgap-secure/test/manifest.txt
```

A given line of this file, which you uploaded to S3 in an earlier step, is passed to each mapper.

12. Next to **Output S3 location**, enter

```
s3://rail-dbgap-secure/test/out/
```

This is where the number of samples in which each k-mer appears will be written.

13. In the **Arguments** box, enter

```
-inputformat org.apache.hadoop.mapred.lib.NLineInputFormat
```

to tell Hadoop that each mapper should be passed a single line of the input file `manifest.txt` . The box in which you're entering data should now look as depicted below.

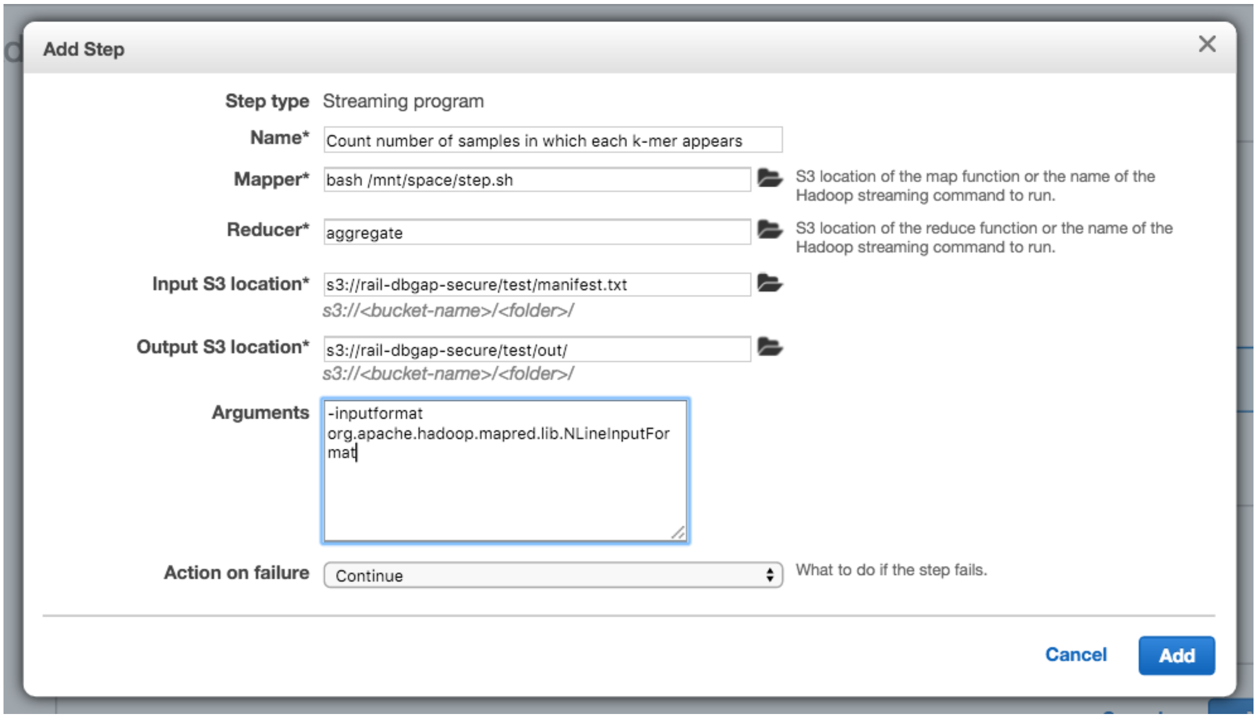

14. Click **Add**. The **Software Configuration** step is now finished and should look as follows.

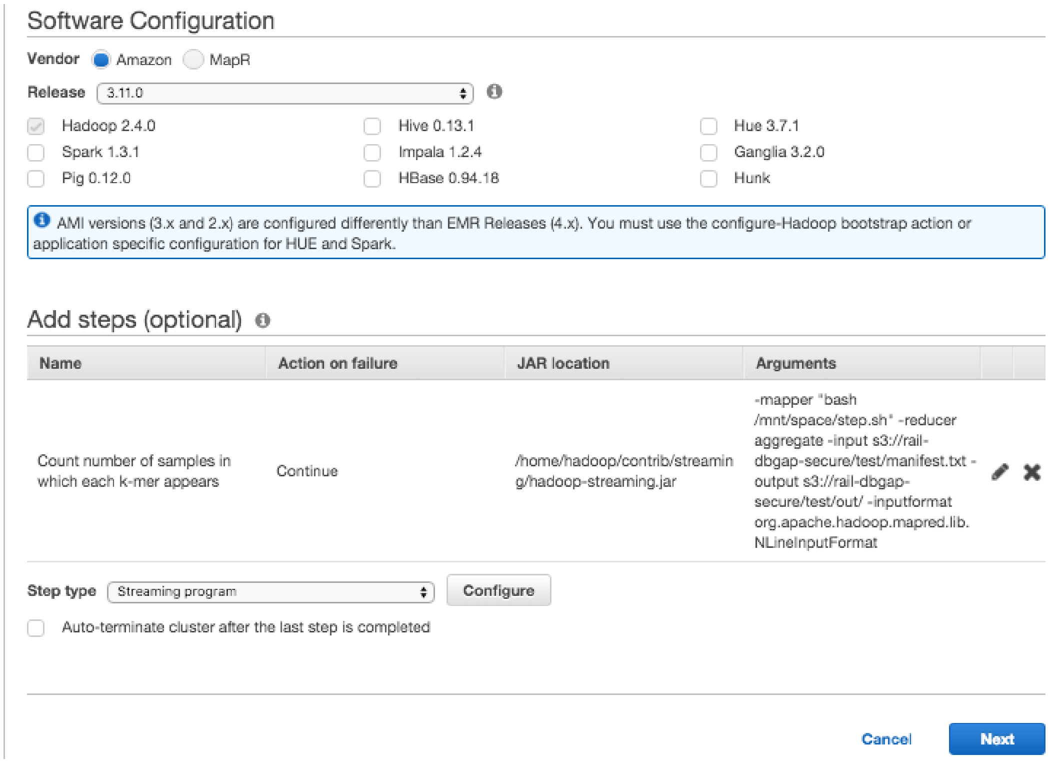

Now click **Next**. Under **Hardware Configuration**, click the **Network** drop-down menu, and select the VPC that does not have **(default)** next to it. This is the VPC that was created as part of the secure CloudFormation stack during AWS setup.

15. Select 1 `m3.xlarge` for the master EC2 instance group, and 1 `m3.xlarge` for the core EC2 instance group.
16. Click **Next**. Under **Logging**, enter the S3 folder `s3://rail-dbgap-secure/logs/` to ensure that logs end up in the secure bucket created as part of the CloudFormation stack during AWS setup. Keep **Debugging** selected, but deselect **Termination protection**; turning this option on would simply prevent termination of the EMR cluster from the API or the command line.

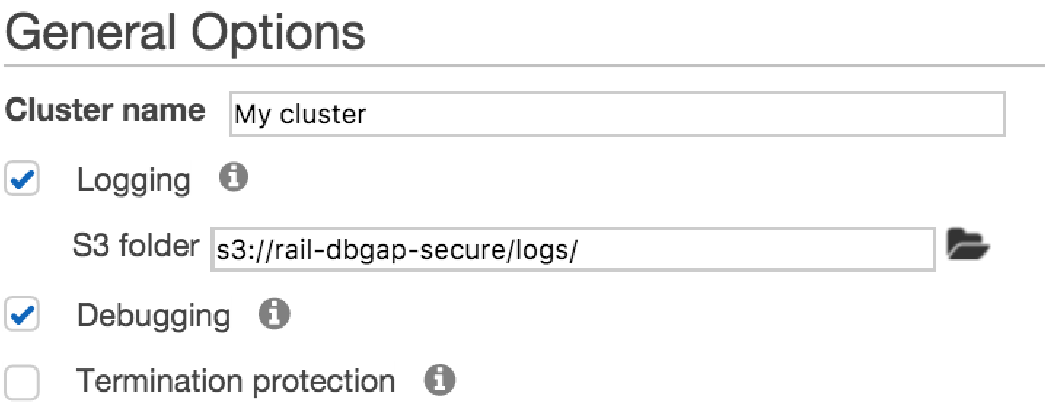

17. Next to **Bootstrap Actions**, select **Custom action** from the drop-down menu, and click **Configure and add**, Enter the **Name** "Encrypt local storage", and specify the **JAR location** `s3://rail-emr/encrypt_local_storage.sh` , which is exactly the script here. As described in the Rail-dbGaP protocol specification, this script uses Linux Unified Key Setup (LUKS) to create an encrypted partition with a randomly generated keyfile, where temporary files, the Hadoop distributed file system, and buffered output to the cloud storage service are all configured to reside on the encrypted partition via symbolic links.

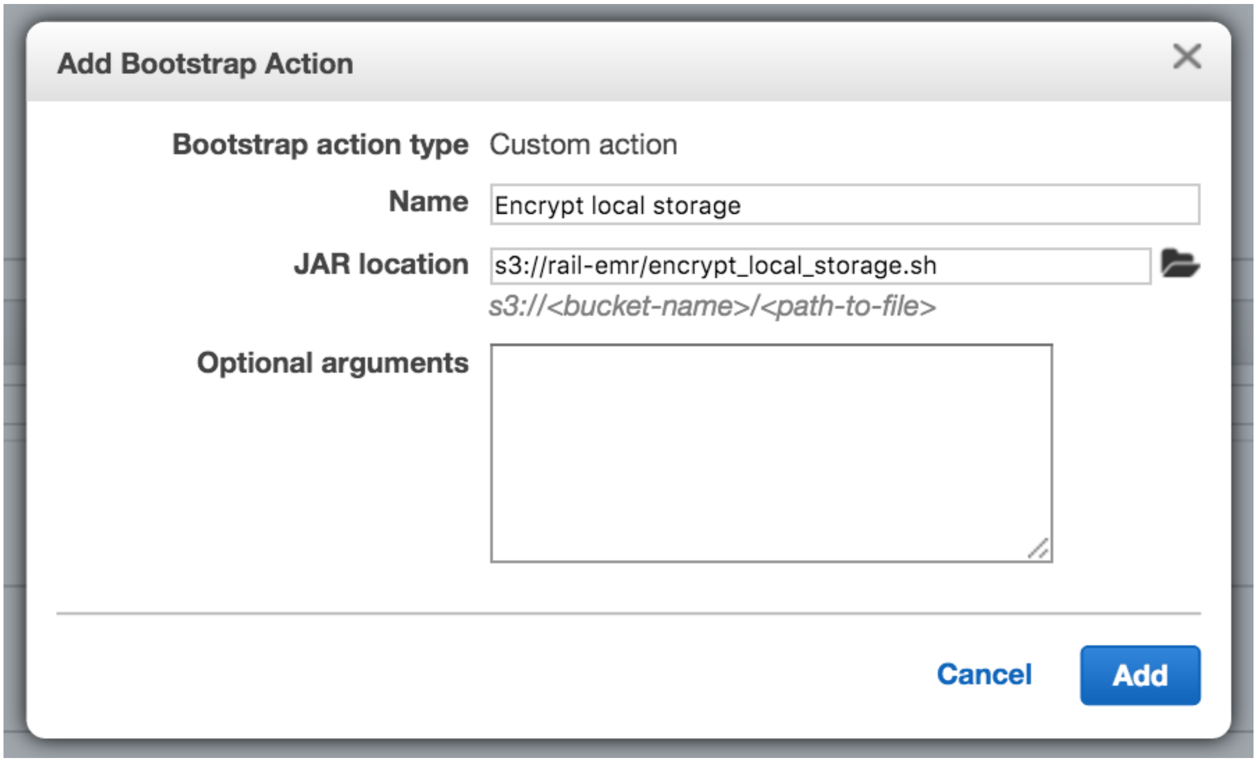

18. In exactly the same way, configure bootstrap actions to obtain the four bootstrap actions depicted below. (Note that these four bootstrap actions should be in exactly this order.)

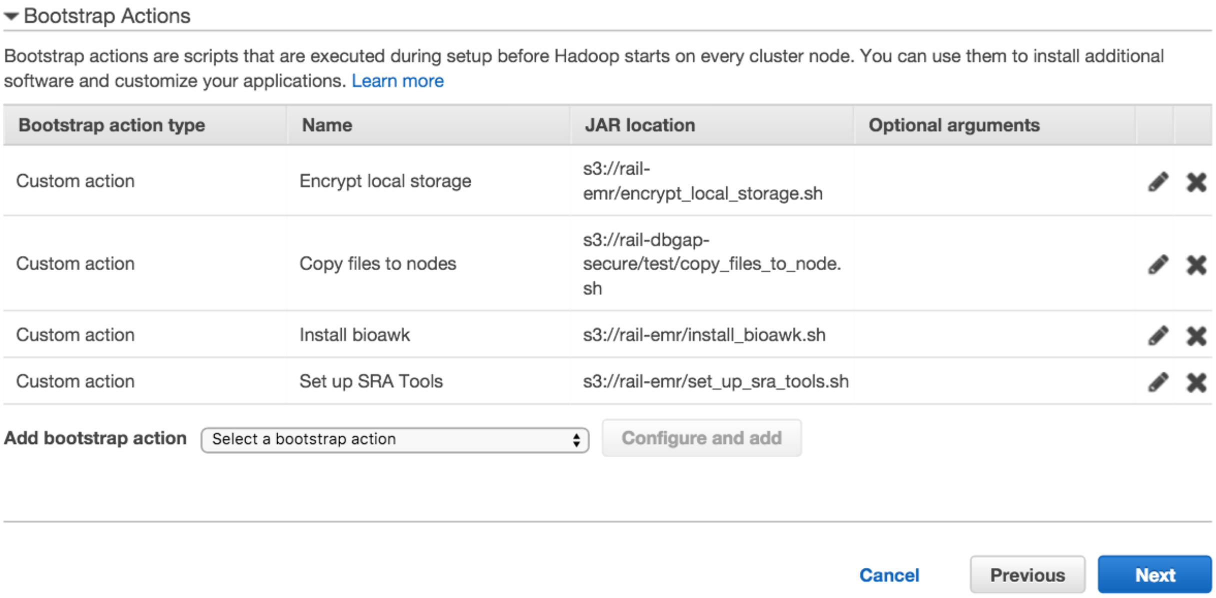

`install_bioawk.sh` just installs bioawk, and `copy_files_to_node.sh` is the script you created in a previous step that securely copies the dbGaP key and step script on S3 to each node of the EMR cluster. `set_up_sra_tools.sh` downloads and installs SRA Tools, which includes `fastq-dump`. The script also configures a workspace on the encrypted partition using the dbGaP key so that `fastq-dump` can download samples from the dbGaP project protected by the key.

19. Click **Next**. Under **Security Options**, make sure **Proceed without an EC2 key pair** is selected in the drop-down menu. This prevents SSHing to the cluster.
20. Under **EC2 security groups**, for **Master**, select the security group with the prefix `dbgap` and `EC2MasterSecurityGroup` in its name; for **Core & Task**, select the security group with the prefix `dbgap` and `EC2SlaveSecurityGroup` in its name. (A warning may appear while you're performing these tasks; it'll disappear once both security groups are selected.) These security groups prevent connections that originate from outside the cluster. EMR automatically pokes holes in these security groups to accommodate only essential web services.
21. Under **Encryption options**, select **S3 server-side encryption** from the drop-down menu next to **S3 Encryption (with**

EMRFS). This enables encryption at rest on S3 by setting the EMRFS configuration parameter `fs.s3.enableServerSideEncryption=true` .

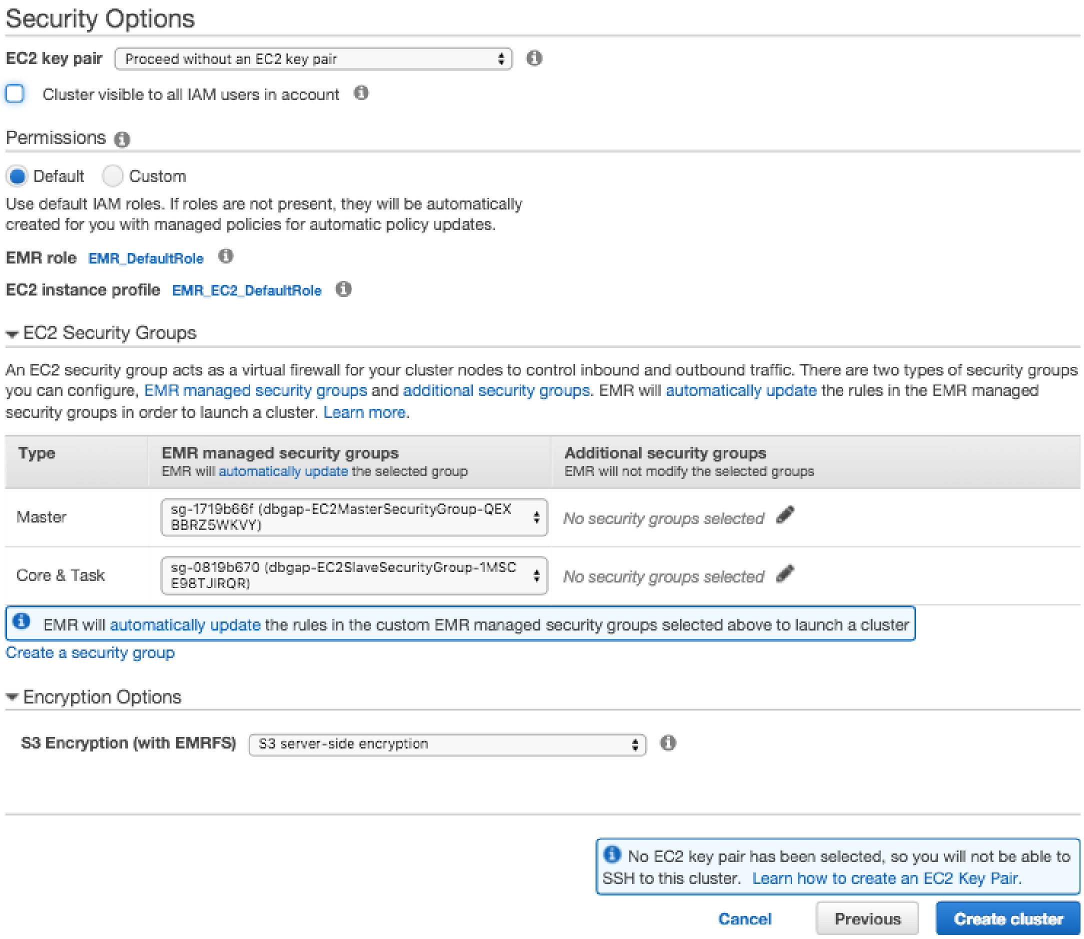

22. Click **Create cluster**. The EMR interface for monitoring your job flow will appear. Wait for the job flow to complete.
23. After the job flow is finished, the EMR interface should look as follows.

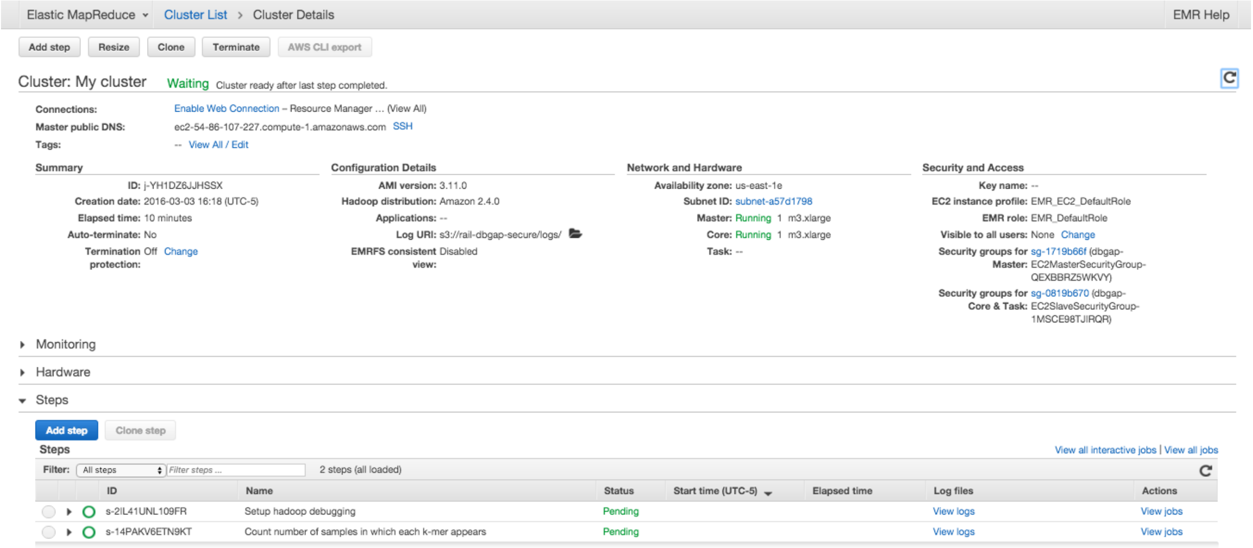

Click **Terminate** to terminate the cluster. Now click **S3** in the AWS console. Navigate to the folder `s3://rail-dbgap-secure/test/out` . The output text files in this directory list k-mers and the number of samples in which each k-mer was found. You can click to download them. *If these data weren't test data and were actually dbGaP-protected, you would not be allowed do this unless your local device were authorized to store the data.*

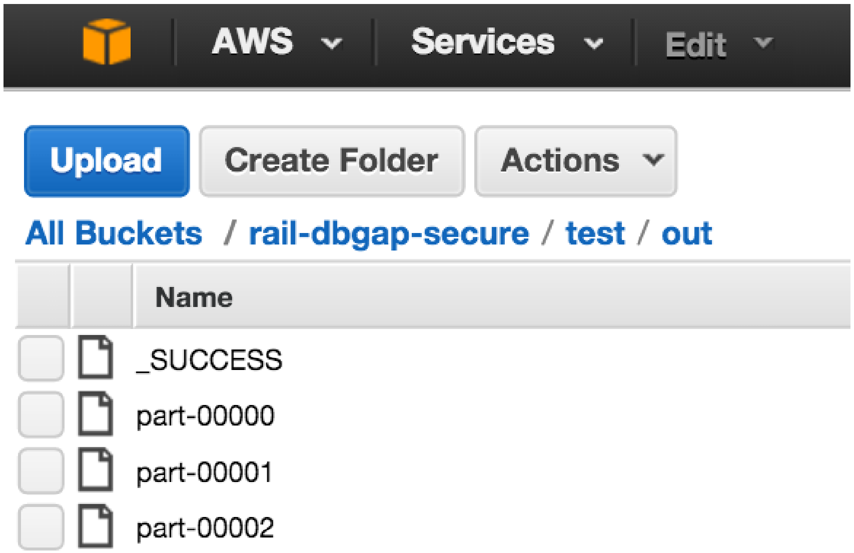

A note on TCGA and other data sources

While we do not provide explicit instruction on how to download TCGA data, the user may substitute the SRA Tools bootstrap and `fastq-dump` for analogs that use CGHub's GeneTorrent or the Seven Bridges API. Similar substitutions apply to other protected data sources. No other parts of the Rail-dbGaP protocol need modification.

Helpful notes for administrators

As for any new AWS account, you should consider how you would like to configure billing. Consolidated billing can be convenient if you are managing multiple AWS accounts simultaneously.

Further, you should consider raising your EC2 instance limits. This is particularly important if you plan to analyze large datasets (more than 100 RNA-seq samples at a time). To raise your limits, visit this page.
