# Supplementary material for: Rail-dbGaP: analyzing dbGaP-protected data in the cloud with Amazon Elastic MapReduce
Source: Bioinformatics. 2016 Apr 21;32(16):2551–3. doi: 10.1093/bioinformatics/btw177 (PMC4978928; doi:10.1093/bioinformatics/btw177)
Supplement: Supplementary Data [file supp_btw177_rail-dbgap_supplement.pdf]

SUPPLEMENTARY MATERIAL

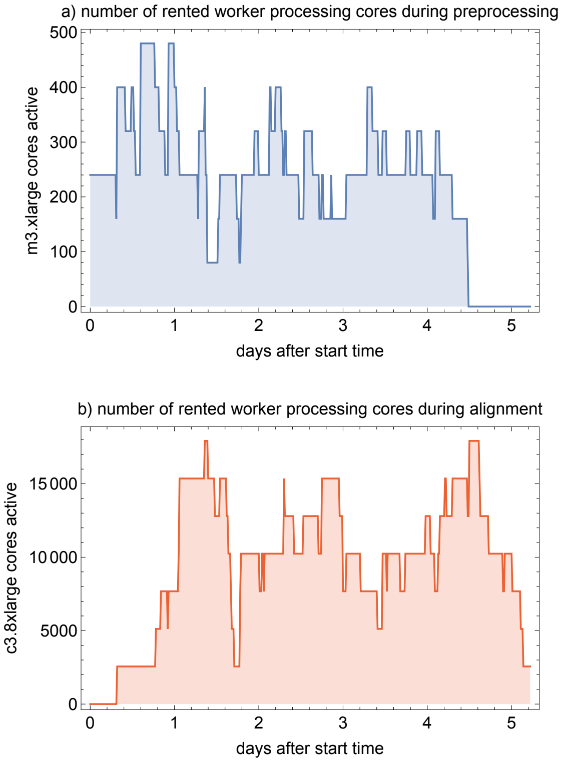

Fig. 2. The number of rented worker cores across clusters running a) preprocess and b) alignment job flows for the duration of all job flows.

3.1 Computation details

We analyzed 9,662 RNA-seq samples from the V6 release by the GTEx consortium. These were divided randomly into 30 batches of approximately the same size: two batches had 323 samples, and the others had 322 samples. Rail-RNA splits analysis of each batch up into a preprocess job flow, which securely downloads raw reads from dbGaP and uploads preprocessed versions to S3; and an alignment job flow, which aligns preprocessed reads and writes results back to S3. Preprocess job flows were run on 21 m3.xlarge Amazon EC2 instances, each with four 2.4 GHz Intel Xeon E5-2670 v2 (Ivy Bridge) processing cores and 15 GB of RAM. Alignment job flows were run on 81 c3.8xlarge Amazon EC2 instances, each with 32 Intel Xeon E5-2680 v2 (Ivy Bridge) processing cores and 60 GB of RAM. One instance of every EMR cluster was a master and the rest were workers, so up to 80 worker processing cores were active for each preprocess job flow and up to 2560 worker processing cores were active for each alignment job flow. All EC2 instances were obtained from the EC2 spot marketplace, which allowed us to reduce our cost to the fluctuating market price, typically a fraction of the standard (on-demand) price. Job flows were submitted manually, with preprocess job flows staggered to minimize issues with simultaneous downloads from the dbGaP server. The number of rented worker cores across the 5 days, 5 hours, and 28 minutes during which job flows were run is summarized in Fig. 2. We typically could not download more than 240 samples at once across all our preprocess job flows without sustaining failures; jumps above 240 active cores in the activity for preprocess job flows depicted in Fig. 2 point to inactive cores on EMR clusters waiting for hanging tasks to complete. Rail-RNA users can expect to observe similar behavior. Mapped read proportions across GTEx are depicted in Fig. 3.

3.2 Cost calculations

We used the AWS Cost Explorer to sum costs across the five days over which the computation ran: November 30, 2015 through December 4, 2015. The total cost of our analysis was US$28,368.15, which gives an average cost of US$0.32 per 10 million reads aligned. Raw cost data downloaded from the AWS Cost Explorer are available at https://github.com/nellore/runs/blob/master/gtex/costs.csv. Costs broken down by AWS service are depicted in Fig. 4.

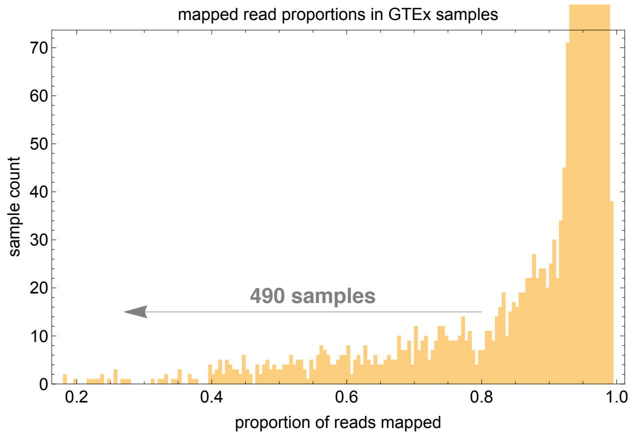

Fig. 3. Mapped read proportions across 9,662 GTEx RNA-seq samples from Rail-RNA alignment job flows. 490 samples each had fewer than 80% mapped reads.

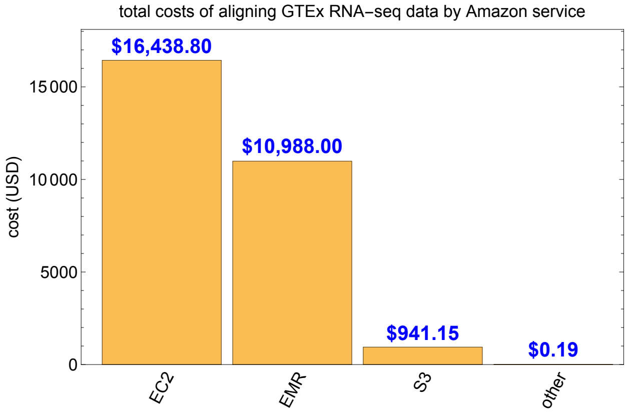

Fig. 4. Total costs of GTEx analysis jobs divided up by Amazon service. The trivial contributions of the “other” costs are from Simple Queue Service (SQS) and SimpleDB.
